# Alkenyl Succinic Anhydride: The Question of Covalent Bonding and Chemistry Considerations for Better Sizing—Review

**DOI:** 10.3390/polym15132876

**Published:** 2023-06-29

**Authors:** Yao Ntifafa, Lebo Xu, Sara Bollinger, Yun Ji, Peter W. Hart

**Affiliations:** 1 WestRock, 2742 Charles City Road, Richmond, VA 23231, USApeter.hart@westrock.com (P.W.H.); 2 Department of Chemical Engineering, University of North Dakota, Grand Forks, ND 58202, USA

**Keywords:** Alkenyl Succinic Anhydride (ASA), cellulose, paper sizing, covalent bonding, sizing agent stability, sizing agent retention

## Abstract

Alkenyl Succinic Anhydride (ASA) is a sizing agent used in papermaking to increase the water repellency of paper. Almost 60 years after the introduction of the chemical in papermaking, scientists still have differing views on how ASA interacts with cellulose. Several experiments were conducted to bring more clarity to the ASA sizing mechanism, especially on the contentious question of ASA-cellulose covalent bonding or the esterification reaction between ASA and cellulose during papermaking. Herein, research papers and patents, including experiments and results, from the 1960s to 2020 were reviewed. Our investigation revealed that the ester bond formation between ASA and cellulose is insignificant and is not a prerequisite for sizing effectiveness; the main ASA-related material found in sized paper is hydrolyzed ASA or both hydrolyzed ASA and ASA salt. In addition, ASA emulsion stability and ASA emulsion retention are important for sizing efficiency improvement.

## 1. Introduction

**Figure 1:**
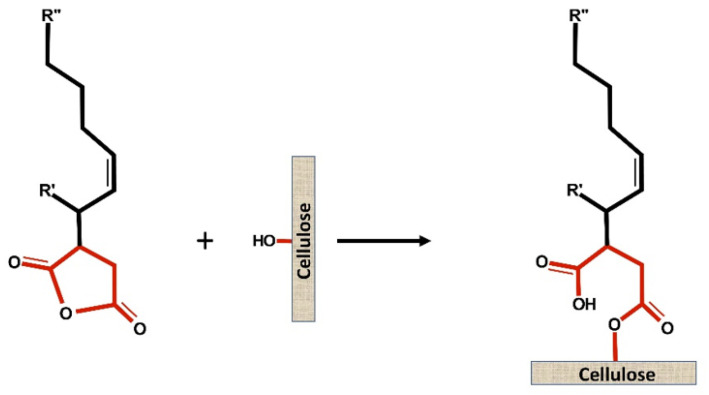
Scheme of ester bond formation between ASA and cellulose.

The objective of paper sizing is to delay wetting by reducing the fiber absorbency. Alkenyl Succinic Anhydride (ASA) was introduced as a sizing agent in papermaking in 1963 by Wurzburg and Mazzarella [1]. ASA is an organic compound with cyclic dicarboxylic anhydride and a tetrafurandione (Figure 1). The chemical is light yellow color, oil-like, non-soluble in water, and liquid at room temperature [2,3].

Today, common internal sizing chemicals (e.g., alum/rosin, AKD [Alkyl ketene dimer] and ASA) are introduced into a pulp slurry at the wet end during the papermaking process. Alum/rosin sizing is commonly used for acidic papermaking with a typical pH range from 4.0 to 5.5. In this pH range, alum forms the right aluminum species to either react with soap rosin or to retain dispersed rosin (DRS) for sizing development. AKD and ASA are used in alkaline papermaking processes, where ASA has a higher reactivity to cellulosic fiber compared to AKD. Moreover, ASA has also been reported to be applied in acidic paper-making conditions [4,5,6,7,8].

Due to the insolubility of ASA in water, ASA oil needs to be emulsified before adding to the wet end for homogenous distribution in pulp slurries. The emulsifier can be starch-based (cationic starch) or polymer-based (polyacrylamide). Typical ASA emulsion particle size is around 1 µm. The ASA emulsion has a short shelf life, and thus the chemical is typically emulsified on-site in the mill and dosed to the wet end as soon as possible. The emulsion particles are retained in the forming paper sheet at the wet end and forming section. Sizing development mostly happens in the dryer section, where the particles break down to release ASA in contact with the fibers [9,10].

**Figure 2:**
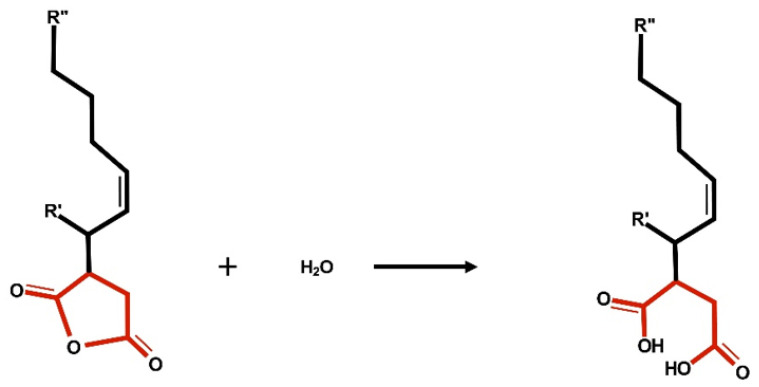
Scheme of the formation of hydrolyzed ASA.

The proposed and widely cited ASA sizing mechanism is the formation of an ester or covalent bond between ASA molecules and fibers. Figure 1 illustrates the ester bond formation between ASA and fiber (cellulose). However, during the last 60 years of papermaking research, this popular mechanism has been questioned. Several scientists are still uncertain about the binding mechanism between ASA and cellulose. In addition, most scientists agree that ASA hydrolyzes in water during the papermaking process (Figure 2) [7,11,12,13].

The covalent bonding question becomes a heated debate between scientists who support the ester bond formation as the primary sizing mechanism and scientists who believe ester bonding is almost nonexistent and does not play a major role in paper sizing. Due to the confusion around the covalent bonding questions, many authors prefer to use the expressions “widely accepted”, “generally accepted”, “generally understood”, “traditionally”, or “assume” in reference to the ASA-cellulose covalent bond formation [7,12,14,15,16,17,18,19].

In this review, patents and research papers with experimental data were reviewed to understand the ASA sizing mechanism since 1963. In addition, chemistry considerations for better application of ASA are briefly discussed.

## 2. Debate on ASA Sizing Mechanism in Papermaking

Prior to the introduction of ASA to the papermaking process in the 1960s, the additive was used in the textile industry to impart water repellency to cellulosic fabrics. Patent 2,903,382 by Robert Berls [20] in 1959 provided details on how to make hydrophobic cellulosic fabric using ASA. Different types of ASA with chains from 19 to 35 carbon atoms were dissolved in solvent such as isopropanol, benzene, toluene, chloroform, carbon tetrachloride, ammonia, morpholine, and water emulsions. The fabric was dipped into the resulting solution then heat cured. The recommended ASA concentration was from 0.7% to 2.5% (14 to 50 lb/t) of the weight of the fabric. The spray test method of the American Association of Textile Chemists and Colorists A.S.T.M. Designation: D583-54 was used to evaluate water repellency. There was no mention of esterification reaction nor covalent bonding in the claim. In addition, the ASA application is like wax application to textile fiber for hydrophobicity, as claimed in patent 2,759,851 in 1956 by Fluck, Pluckemin, and Logan [21].

In 1963, Wurzburg and Mazzarella [1] mixed ASA with different derivatives of starch to form an emulsion. It was claimed that the application of the ASA emulsion on fiber made the sheet hydrophobic. The patent demonstrated the sizing process with the use of the ASA emulsion and the addition of alum, aluminum chloride, long chain fatty amines, sodium aluminate, polyacrylamide, animal glue, polyamide polymers, primary amine starch derivatives, secondary amine starch derivatives, tertiary amine starch derivatives, and quaternary amine starch derivatives.

The author recommended to use 0.5 to 2 parts by weight of cationic starch per 1 part of the sizing agent to obtain adequate results. Different dosages from 0.25% to 2% (5 to 40 lb/t) of the sizing agent were used in 14 different examples to support the claim. Uranine dye and ink dip tests were used to prove the hydrophobicity of the sheets. There was no disclosure of covalent or ester bonding between ASA and cellulose in the patent document.

Cuculo [22] at NC State University tried to esterify succinic anhydride with cellulose in 1971. First, successful results were obtained from the reaction between viscose rayon cellulose and succinamic acid. The samples were baked in succinamic acid at 136 °C, 183 °C, and 207 °C, water-washed, and then treated with 3% sodium sulfate to form sodium cellulose-hemisuccinate. The degree of substitutions for the reactions were, respectively, 0.03, 0.24, and 0.25. The author concluded that the degree of the substitution of reaction depends strongly on the reaction temperature. Second, when succinic anhydride in water was used under comparable conditions to those of the succinamic acid, the author stated that there was no evidence of ester formation. The reported reaction yield with succinamic acid was 36%, and the author mentioned that ammonia copiously evolved during the reaction. The recommended temperature using the succinamic acid is above 150 °C.

McCarthy and Stratton [23] studied the reaction between cotton linters pulp and ASA in 1986. In one study, the cotton linters and ASA were reacted in N,N-dimethyl formamide with triethyl amine as a catalyst. In another study, the cotton linter pulp (washed in chloroform-ethanol solution for 48 h and air dry for several hours) and a high concentration of ASA (1.5% or 30 lb/ton) were reacted. Poly(1,2-dimethyl-5-vinylpyridinium bromide), or DMVPB, was used as ASA retention aid. In both studies, FTIR data showed the formation of ester bonds, however the efficiency of these reactions was not reported. In McCarthy’s thesis in 1987 [24], the author showed by FTIR that ASA reacted with ethanol to form ester bonds. It is not known if part of the ASA formed ester bonds with ethanol rather than cellulose since the pulp was washed 48 h with chloroform-ethanol solution.

Wan [25] studied the mechanism of ASA sizing in 1988 using C 14-labelled tetradecenyl succinic anhydride (TDSA) and tetradecenyl succinic acid (TDSAcid). TDSA and TDSAcid emulsions were made using starch as an emulsifier at a 1:3 ratio. The emulsions containing 1.3% total solid was charged to the pulp to make 60 g/m^2^ hand-sheets. The C14-labelled technique was used to quantify the ASA component in the sheets after chloroform extraction. The results showed that unreacted ASA is predominantly found in the sheet and about 25% of ASA can produce sizing and is not extractable with chloroform.

According to the author, the retained ASA could be explained by covalent bonding due to three reasons. First, the continuous increasing in sizing at room temperature suggests the reduction in moisture allows the ASA to react with the hydroxyls of cellulose. Second, the ASA molecule, which is under constant reorientation during the sizing process, would undergo hydrolysis if at any time the hydrophilic part of the molecule is exposed to moisture. The retained molecule can be assumed to be held by stronger irreversible bond forces. Third, the inability of the ASAcid (hydrolyzed form of ASA in the dicarboxylic acid form) to size hand-sheets demonstrates that hydrogen bonding is not the mechanism of ASA sizing, thus covalent bonding must be the mechanism. The author concluded that this evidence suggests that unextractable ASA is bound to cellulose via covalent bonding. In addition, according to the author, solvent sized sheets gave better sizing than conventional emulsion sized sheets due to the absence of water that induces hydrolysis.

Nishiyama et al. [26] used a series of extraction, impregnation, and cellulase treatment experiments to study the ASA bonding mechanism under common papermaking conditions in 1996. Pulp and ASA-starch emulsion containing 0.2% (4 lb/ton) ASA were used to make 60 g/m^2^ hand-sheets. Chloroform, water-acetone, and dimethyl sulfoxide (DMSO) were used to extract the hand-sheets and cellulase was used to isolate the ASA residues.

The measurement of the extracts by gas chromatography showed that not all ASA was extractable, and the NMR data showed no presence of formed ester linkage between the ASA and the hydroxyl group of cellulose. When an old ASA emulsion, which has no chance to form ester linkages with cellulose, was used, the extraction results also showed not all ASA was extractable. The authors concluded that a small amount of ASA is physically entangled in the cellulose network without forming covalent bonds.

Impregnation of filter papers with acetone solutions of ASA, non-reactive ASAcid, and non-reactive ASAcid methyl esters were also studied. Sizing occurred in all cases, but no ester linkages between ASA and cellulose-OH were found to exist.

To ensure that ester bonds were not formed then destroyed by the cellulase isolation and analytical techniques employed, the tests were repeated using cellulase treatment stable conditions. These conditions ensured any ester linkages between cellulose-OH and ASA would not be cleaved during isolation and analysis. These tests were repeated using sample A which is ASA-alum-sized hand-sheets, sample B which is ASA-PAE-sized hand-sheets, and sample C which is PAE-treated hand-sheets. The residues after the enzymatic treatment were 1.4, 0.7, and 1.1% for the samples A, B, and C, respectively. The analysis of the residues showed the samples A and B contain 10% and 15% ASA, respectively. Further analysis of the residues of samples A and B was conducted; the FTIR data showed that most ASA components do not form ester bonds with cellulose, and the predominant components of ASA in the sheets A and B are in the form of ASAcid. The author concluded after the extraction, impregnation, and cellulase treatment studies that the ASA sizing mechanism should not be explained by the formation of ester linkages.

In 2000, Akira [27] studied the extracts of ASA-sized hand-sheets with ASA content of 2.2 mg/g. Pulp and ASA-starch emulsion containing 0.2% ASA was used to make the 60 g/m^2^ hands-sheets. A series of extractions using water, chloroform, 1% Tween 80 at 20 °C, and 1% Tween 80 at 70 °C were performed. The extracted sheets were analyzed with pyrolysis gas chromatography–mass spectrometry (GCMS). The amount of ASA retained in the sheet was respectively 1.7 mg/g, 0.6 mg/g, 0.3 mg/g, and almost zero for the listed extraction conditions, respectively. According to the author, the extraction results showed that virtually no covalent bonds between ASA and hydroxyl groups of cellulose were present in the hand-sheet. These results also indicated that chloroform is not a suitable solvent to completely extract ASA even though the ASA is present only by physical interactions without forming covalent bond with cellulose.

Moreover, to understand the efficient state of the ASA, three types of hand-sheets containing 0.4% (8 lb/t) ASA were made with different ASA emulsions: fresh emulsion, fresh emulsion and pulp stirred for 3 days, and 3-days-old emulsion. Only the fresh emulsion paper exhibited a sizing effect. SEM data showed large flocs of ASAcid in the two sets of sheets made with either stirred or old emulsion. The set made with fresh emulsion had well dispersed ASA contained within the sheet. The author concluded that fresh ASA emulsion spread over the fiber surface and promotes hydrophobicity.

In 2002, Yu and Garnier [28] concluded from their work that ASA and cellulose are covalently bonded. In the experiment, cellulose film was regenerated from cellulose acetate on glass. ASA vapor was deposited on the substrate in an airtight adsorption cell, which was heated in an oven at a preset temperature and then quenched. The contact angle was measured immediately, and the measurement revealed the substrate was hydrophobic. According to the authors, the presence of the OH groups, the temperature dependence of the reaction, the non-decrease in hydrophobicity (even after chloroform extraction), and the time frame (hour) of the reaction are typical for the formation of covalent bond by esterification.

Acha et al. [29] studied the reaction between ASA and wood flour in 2003. The esterification reaction was carried out by immersing the wood flour with an average diameter of 57 μm in 96 g/L (9.6% or 192 lb/ton) ASA in acetone solution; 4-dimethylaminopyridine was used as a catalyst. The mixture was refluxed at 56.5 °C for 4 h. The product was dried at 70 °C in an oven and later washed intensively with distilled water to remove unreacted reagents before it was dried again at 70 °C under vacuum to constant weight. The esterified product was later mixed with unsaturated polyester (UPE) based on bisphenol A-fumarate cross-linked with styrene for molding; benzoyl peroxide was used as an initiator, and polymethyl methacrylate was used as a thermoplastic modifier.

Diffuse reflectance infrared Fourier transform spectroscopy (DRIFTS), a saponification test, and an acid value test were used to analyze qualitatively and quantitatively the esterified products. The results showed that esterification did occur. The yield was 166 g ASA for 1 kg treated wood flour (16.6%).

In 2006, Wang [30] studied ASA sizing mechanism by comparing FT-IR spectra between ASA-sized hand-sheets, synthesized ASA-cellulose compound, hydrolyzed ASA, hydrolyzed ASA-sodium salt, hydrolyzed ASA-calcium salt, and hydrolyzed ASA-aluminum salt. The amount of ASA charged to the studied hand-sheets was 0.5% or 10 lb/t oven-dried pulp. The results showed no covalent bonding between the ASA and the cellulose. In addition, the results suggested that the sizing material found in ASA-sized hand-sheet was mainly hydrolyzed ASA and its salt. The paper also demonstrated that to size hand-sheets directly with hydrolyzed-ASA emulsion resulted in low sizing degree. Nevertheless, after dipping hydrolyzed-ASA-sized hand-sheets in an alum solution, the sizing degree could be brought up to the level close to ASA emulsion sized paper.

Hundhausen et al. [31] studied the reaction of ASA and particleboard chips in 2010. The objective was to investigate if ASA bonds to the surface of the chips to repel water. The chips were dried to 1% moisture content and then were wetted with 3% or 60 lb/t ASA (based on the oven dry wood). The samples were processed in a rotating reactor at 130 °C and 1013 mbar for 1 h at 12 rpm.

FTIR analysis before and after the treatment revealed that esterification did not occur and both hydrolyzed and non-hydrolyzed ASA were located on the chip surface after curing in the reactor. In addition, FTIR measurement of the chips after Soxhlet extraction did not show any ASA signal, which indicated that the ASA did not esterify and was completely removed by the solvent during the extraction.

Furthermore, ASA-ethyl acetate and ASA dimethyl sulfoxide (DMSO) solutions were applied to veneers. It turned out that the ASA was completely removed after extraction when it was applied in ethyl acetate but not completely in DMSO; the covalently bound ASA could not be extracted. The unextractable amount, or the yield of the reaction, is not known. According to the authors, the ASA esterification with cellulose depends on the solvent that provides certain mobility and orientation to the sizing agent. DMSO leads to esterification but its high boiling point (189 °C) and its poor miscibility with ASA make it disadvantageous. Water emulsions would hamper the ASA penetration into the wood; xylene is a better candidate but harmful for human health.

Masumi et al. [32] used Time-of-Flight Secondary Ion Mass Spectrometry (ToF-SIMS) to study the ASA sizing mechanism in 2012. ToF-SIMS is a surface-sensitive analytical method that uses a pulsed ion beam to remove molecules from the very outermost surface of the sample. ToF-SIMS collected on an ASA-sized paper sample was compared to the spectra collected from ASA, ASA/water mixture, ASA/NaOH solution, cellulose, and synthesized ASA-cellulose compound. The results indicated that the form of ASA in ASA-sized paper was different from that in ASA-cellulose compound, which did not support an ester bonding hypothesis. The ToF-SIMS from ASA/NaOH solution was close to that from ASA-sized paper. This suggested the ASA in ASA-sized paper was likely hydrolyzed ASA or its salt.

In 2015, Li et al. [33] studied the anchorage of ASA on cellulose by dipping cellulose film and filter paper in both acetone-ASA solution and ASA emulsion. The cellulose film was generated by immersing cellulose acetate film in 0.5% sodium methoxide in methanol for 12 h. The film was repeatedly rinsed with running deionized water and methanol, air-dried, and stored in a desiccator. The filter paper used was a commercial ash-free filter paper. The ASA-acetone dipping solution contained 1% (20 lb/ton) of ASA and the ASA emulsion, which also contained 1% ASA, was prepared by using laponite as particle stabilizers after being modified by urea, alanine, tetramethylammonium chloride and melamine or in combination with poly-aluminum sulfate (PAS).

The FTIR and the XPS results showed the presence of covalent bonds between ASA and cellulose. The authors concluded that sizing is due to the formation of the ester bond. The ASA-acetone sizing solution performed better than the ASA emulsion co-stabilized by laponite and PAS due to the absence of water, which tends to produce hydrolyzed ASA more easily. Although the yield of the esterification is not known, the authors suggest that the amount of extractable ASA species in the paper is higher than the amount anchored. In addition, laponite and PAS co-stabilized emulsions lead to high sizing performance but greatly depend on the use of aluminum sulfate. According to the author, the added aluminum sulfate may improve the retention of ASA or anchor the hydrolyze ASA to the cellulose.

Lackinger et al. [34] attempted to provide proof in favor or against covalent binding of ASA to cellulose in 2015. To avoid the interaction of starch, filter paper was soaked in acetone solutions with various levels of ASA (blank, 1, 5, 10, and 25%) and dried under different conditions (room temperature, drum dryer at 115 °C, drum dryer at 115 °C, and oven at 125 °C). The COOH-selective fluorescent labeling for cellulosic material and the gel permeation chromatography (GPC) with a multi-detector setup for covalently bound sizing agent detection approach was used to provide profiles of carboxyl groups along the molecular weight distribution. The result showed that only about 0.5% of the total ASA covalently bonded to cellulose. Furthermore, hand-sheets were made with 0.2% ASA emulsion in starch, and the correlation between the small amount of covalently bonded ASA and the sizing efficiency of the sheets were studied. The results indicated that there was no correlation between the amount of covalently bonded ASA and sizing efficiency. The authors concluded from the two sets of experiments that ester bonds between ASA and cellulose were a small fraction and were not a prerequisite for sizing.

In 2016, Porkert [35] worked on the localization of ASA in the sheet using confocal white light microscopy and the dye Sudan Red 7B. Preliminary experiments using thin layer chromatography and FTIR were conducted to ensure that the dye does not affect the processability and the performance of ASA and ASA emulsions. Dyed ASA dosages of 0%, 0.05%, 0.1%, 0.2%, 0.3%, and 0.4% were applied to 100 g/m^2^ hand-sheets and the shades were correlated to each value. Different sheets were mapped to study the relation between size performance and the homogeneity of the ASA distributions under different dosages and conditions. Some of the conditions are reactive ASA emulsion, cationic and anionic hydrolyzed ASA emulsions, ASA-Starch ratio, and emulsion age.

The study revealed that the ASA sizing mechanism depends on the distribution of the ASA in the sheet. There is a direct correlation between the agglomeration of ASA and sizing performance: the more agglomerates, the weaker the sizing. The application of reactive ASA led to uniform distribution, which results in better sizing performance. Cationic and anionic hydrolyzed ASAs have higher agglomerates thus low sizing performance. Better size distribution and performance were obtained at ASA/starch ratio of 1:1 for low dosage of 0.05% (1 lb/ton) ASA. Concerning the aging, the first 20 min gave the best performance for low and high dosages.

According to the author, the distribution of ASA supports the sizing mechanism based on homogenous molecular distribution and orientation. Hydrophobization is solely based on the physical distribution and orientation of the ASA molecules within the sheet. In addition, the application of reactive ASA is the key element to obtain homogenous distribution as the application of the hydrolyzed ASA led to agglomerations. Furthermore, the esterification reaction between ASA and cellulose is very unlikely to happen during the papermaking process, and the phenomena of size reversion, size reactivation, and size migration exclude any significant extent of esterification but are only explainable by intra- and inter-molecular mobilities.

The objective of Wulz [36] in 2020 was to hydrophobize the surface of paper by vapor deposition of ASA, palmitoyl chloride, TFAA/Ac2O (trifluoroacetic anhydride/acetic anhydride), and TFAA/Acetic acid mixtures and hexamethyldisilazane (HMDS). Unsized and untreated papers were used for the experiment. The papers were stored at 23 °C and 50% humidity for at least 24 h. The gas phase ASA deposition was performed at 50 °C and 100 °C at 20 mbar for 2 h. The experiment with ASA did not lead to hydrophobicity, however the experiment with other additives led to hydrophobicity via formation of ester bonds. Due to the poor hydrophobization of the ASA gas phase deposition, no further research was carried out with ASA, and no FTIR data were collected.

There is no doubt that ASA sizes paper. However, the sizing mechanism, especially the covalent bond formation between the ASA and cellulose is a divergence point among scientists, researchers, and papermakers. The covalent bond theory is often used by vendors to explain sizing development. However, the assessment of the available data over the last 60 years shows that the formation of covalent bond is insignificant in ASA-sized paper. Hydrolyzed ASA and or ASA salts are the fundamental elements found in sized paper. Catalysts and or organic solvents can be used to initiate esterification, but such conditions are unrealistic in papermaking.

## 3. Overview of the ASA Mechanism

A summary of the proposed reaction mechanisms for ASA sizing suggest that multiple potential pathways exist. The first proposed sizing mechanism is bonded ASA molecules to cellulose via esterification. Though some research using organic solvent, high temperature, or catalyst showed the evidence of ester bond formation, most studies conducted close to commercial papermaking conditions yielded scientific evidence supporting hydrolyzed ASA as the major sizing material in a sized paper. However, the direct application of hydrolyzed ASA to the pulp or the application (coating) of hydrolyzed ASA to the paper does not achieve sizing. Thus, the type of sizing material and the uniform distribution or structure of the sizing material significantly contributes to sizing development [26,27,35,37].

**Figure 3:**
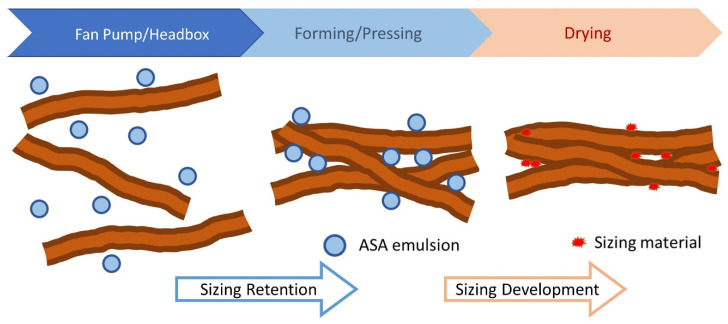
Scheme of ASA sizing process.

Figure 3 shows the main steps of the ASA sizing process; the oil (ASA) is first emulsified and then applied to the pulp. The pulp is drained and pressed to form a sheet. Sizing is developed after the sheet is dried.

In commercial paper mills, common ASA application consists of preparing the ASA emulsion, which is later added to the pulp at a point close to the fan pump. The mixture is moved to the headbox where the average consistency of the pulp is about 1%, and the predominant component is water, not organic solvent. Although other materials such as alum, PAE (Polyamide Epichlorohydrin), GCC (Ground Calcium Carbonate), CPAM (cationic polyacrylamide), etc., are added to the furnish depending on the paper grade and the papermaker, the predominant component remains water, and the chance of the formation of hydrolyzed ASA is high. The slurry is dewatered then dried in the dryer section of the machine to develop sizing, i.e., the resistance to liquid penetration of the sheet (Figure 3).

The commercial papermaking process is more complex than laboratory hand-sheet making, and the chance of covalent bond formation is even lower than the already low laboratory results due to the following reasons:

- Laboratory water quality is superior to that in the mill (deionized water vs. white water) [35,38,39].
- Laboratory ASA dosage is usually higher than that used in the mill, [23,30,31,33,34,35].
- In some studies, ASA is applied in organic solvents (DMVPB, DMSO, acetone, toluene, chloroform, ethanol, xylene, ethyl acetate…) that are not found on paper machines to create idealized systems [23,30,31,33,34,35].
- One additive (ASA) is usually studied in the laboratory to prevent the influence of other variables while in real papermaking processes many organics, inorganics, additives and even bacteria compete with ASA to dwell with the fiber. In short, there are more uncontrolled variables in field experiments than in lab ones [39,40].
- Lastly, in laboratory studies, sometimes the pulp or the substrate has a special treatment not found in the mill. Such treatment can be ethanol or methanol wash of the pulp or the substrate, although it is well known that these solvents esterify with ASA, and their leftovers in the pulp can mislead the interpretation of FTIR results [23,26,33,41,42].

The covalent bond theory was not believed or developed or affirmed by the inventors Wurzburg and Mazzarella in the available patent document [1]. It is assumed that if a strong catalyst, a special condition, or an aid that promotes covalent bonding between the ASA and the cellulose is added to the process in the paper mill, it might be possible to form covalent bonds between ASA and cellulose. Some of these catalysts are triethylamine, methanesulfonic, sulfuric acid, and 4-dimethylaminopyridine [23,26,29,43]. As of now, such aids are missing in paper mills and the amount of covalent bond between ASA and cellulose is very low. Covalent bonding is not a prerequisite and does not play a major role in the sizing. In addition, the contact between ASA and starch in a real papermaking process is far more intimate and extensive than the contact with cellulose [26,34,44]. The “nebule” of starch that vehiculates ASA on the paper machine will eventually break and release the additive. Assuming starch does not evaporate after the burst, which is realistic, another selectivity mechanism will be needed to explain the reason why ASA chooses to bond with cellulose instead of starch, which is in closer contact for the assumed esterification reaction. Furthermore, even if paper mills start using strong catalysts or solvents or a special condition for ASA cellulose esterification, careful choice of these materials needs to be made with respect to the reaction selectivity, so ASA “droplets” enveloped in starch (emulsion) do not react with the starch itself or lignin (coniferyl alcohol and sinapyl alcohol) or other additives or residual wood extractives (fatty alcohols) in the pulp [45,46,47,48] but mainly with cellulose and hemicellulose.

After careful review of the past research efforts both supporting and refuting ester bonding of ASA to cellulose, it is believed that, under routine papermaking conditions, the esterification level is almost null in the papermaking process. Future research efforts need to find aids such as harmless catalysts or solvents that promote better yield of esterification, especially in water and on commercial paper machines. Higher esterification yield should be beneficial for the paper sizing process. We believe high yield covalent bonding equates to strong chemical bonds, which will reduce certain amounts of size reversion due to migration, re-orientation, or fugitive ASA molecules.

## 4. Chemistry Considerations for Better ASA Sizing Application

Sizing performance is a function of size distribution, retention, and development. For an ASA sizing program, though there was debate on the sizing development mechanism, a few researchers agreed that better ASA emulsion stability and higher retention improves sizing results [27,30,37,49,50,51]. Some chemistry considerations for better ASA stability and retention are provided herein.

- Emulsion Stability (better distribution)

ASA emulsion is made from ASA (oil) and an emulsifier such as cationic starch or polymer in water. Typically, concentrated emulsifier is first diluted with process water to a targeted low concentration. Then, ASA oil is introduced to make the emulsion in high shear equipment. When cationic starch is used as an emulsifier, the most efficient size response is obtained when the mass of the starch is up to 6 times the mass of the ASA; this range obviously depends on the paper grade that is being made and other additives that are being used. In general, it is recommended to keep the mass ratio of the cationic starch to ASA at 4:1 and the particle size about 1 μm [12,35,44,52,53].

pH and temperature of the process water can affect the stability of ASA emulsion. It is known that ASA is hydrolyzed rapidly at alkaline water pH. The hydrolyzed ASA has been demonstrated to agglomerate, leading to inefficient distribution of the sizing agent [26,30]. Therefore, maintaining the process water pH at neutral or a slightly acidic range can ensure stable emulsion particle size and better distribution. Alum, citric acid, and adipic acid are generally used to adjust pH [12,14,53,54,55]. Higher temperature generally accelerates chemical reactions, including ASA hydrolysis. If the machine temperature is high, it is better to add ASA emulsion close to the headbox to minimize contact time. In addition, a mill using on-site cooked starch as an emulsifier should pay attention to the starch temperature going to the ASA emulsion process. The optimal temperature for the emulsification process should be determined for better sizing response [2,50,56].

Conductivity is another important water parameter to consider for emulsion (colloidal) stability. High conductivity, due to high electrolyte concentration, collapses the starch or the polymer chain in aqueous solution and leads to unstable emulsion, which in turn leads to poor size distribution [57,58]. The impact of the ionic strength of the process water on the emulsifier can happen at different locations such as the emulsifier dilution point or the ASA emulsion dilution point (push water). For example, if ground water is used for emulsifier dilution, depending on mill location, high hardness (Ca^2+^ or Mg^2+^) or salinity may impair the emulsifier. Pulping soda carry over is a typical ion source for a virgin machine. Recycle machines may experience unexpected conductivity shock due to salt coming with recycled material.

- Emulsion Shelf Life

The shelf life of ASA emulsion is another important factor to control after the ASA-emulsifier ratio, pH, temperature, conductivity, shear, and other water quality parameters are in their appropriate ranges. In general, the emulsion is used as soon as possible, or less than an hour after it is formed, to prevent the hydrolysis of ASA. Several studies have shown that 20 to 30 min is the best shelf life of an ASA-starch emulsion [6,9,35,59,60]. The emulsion starts forming large aggregates after 30 min and becomes hydrophilic; the hydrolysis of ASA accelerates and the use of such emulsion forms agglomerates on the paper surface, which lead to inefficient sizing. Knowing the shelf life also gives the ability to estimate how far from the headbox the emulsion must be injected into the pulp for better sizing performance.

ASA sizing certainly reduces the paper tendency to absorb liquid; however, the short shelf life of the ASA emulsion is one of its application drawbacks. There are some novel chemistries to stabilize and extend the emulsion shelf life. Anionic polyacrylamide (APAM), maleated sunflower oil high oleic (MSOHO), urea, combination of laponite and polyaluminum sulfate (PAS), combination of chitosan and montmorillonite, ethyl oleate succinic anhydride (EOSA), methyl oleate-succinic anhydride (MOSA) and lauric arginate/cellulose nanocrystal nanorods (LAE/CNC) can extend the emulsion shelf life from several hours up to a day. More information is needed on large scale availability for industrial use of these products, their environmental impacts, and side effects on the papermaking process [9,60,61,62,63,64,65,66].

The decrease in sizing performance versus the age of ASA emulsion is known [67] but how the change in the ASA-emulsifier ratio, pH, temperature, conductivity, and shear of the emulsion individually or collectively affects the aging process is not known. More work needs to be done to understand key factors and at what proportion they influence the rapid ageing or the short life of an ASA emulsion.

- Emulsion Retention

The retention of ASA emulsion was reported mainly based on charge attraction mechanism between anionic cellulose, amphoteric ASA emulsion particles, cationic polymers, and cationic aluminum compounds. The ASA molecule and the fiber surface are negatively charged while, at the proper pH, the aluminum species supplied by the alum and PAC are positively charged. In addition, the use of alum or PAC puts more cationic charge on the ASA emulsion, which is easily retained on the anionic fiber surface. These two additives improve the sizing due to higher retention, better anchoring, and alignment of ASA to the fiber [52,68,69].

When properly used in papermaking, alum improves retention, drainage, neutralizes anionics, controls pH, and improves runnability. PAC and sodium aluminate are also used as sources of aluminum ions. PAC is known to size at higher pH, improve sizing efficiency, control pitch, reduce or eliminate barium sulfate (BaSO_4_) deposits, while sodium aluminate is known to size at higher pH with less corrosion, and increases retention and paper strength. Alum and PAC are commonly used for ASA applications; in addition, alum is suitable for acidic paper making, while PAC can be used in neutral or alkaline paper making [70,71,72,73].

**Figure 4:**
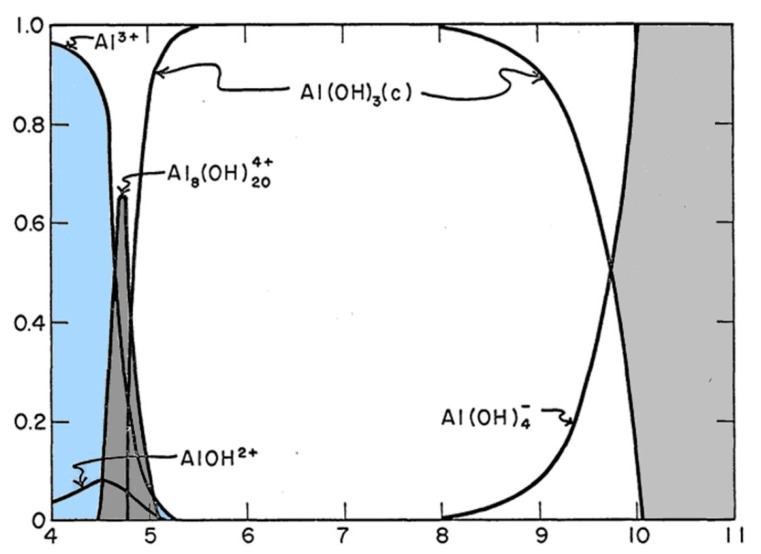
Distribution of Hydrolyzed Aluminum (III) as a Function of pH. Al (III) = 1.0 × 10^−3^ M, 24 h (Adopted from Rubin and Hayden [76]).

Alum was first used in paper sizing in combination with rosin in 1807 by Moritz Friedrich Illig. The chemical was later used in AKD and ASA sizing applications. Alum is known to form a bridge between cellulose and the sizing agent depending on the pH and the species of the aluminum ions available. As shown in Figure 4, alum generally will exist in one of three dominant species: Al^3+^ (pH less than 4.3), Al_8_(OH)_20_^4+^ (pH 4.3–5.0), or Al(OH)_3_ (pH 5.0–8.0). The adsorption of the aluminum ions Al^3+^ to the fiber occurs at low pH and understanding the pH regions of alum is paramount for ASA addition and retention [8,57,74,75].

ASA salt, mainly aluminum-ASA salt, is one of the predominant forms of ASA found in the sheet beside the hydrolyzed ASA. The use of alum enables the formation of the non-tacky aluminum salt which is preferred over the sticky calcium- or magnesium-ASA salt. In addition, the use of alum controls the deposit of hydrolyzed ASA, and the aluminum salt of the hydrolyzed ASA may interact with the anionic charges on cellulose to improve sizing. Furthermore, the application of alum can anchor free hydrolyzed ASA to the fiber as a metal salt to reduce, to delay, or to reverse size reversion due to migration, reorientation or fugitivity [26,52,67,75].

The dosage of alum or PAC is critical as it can improve or impair the papermaking process and paper properties. It is reported that 0.5% alum based on dry pulp is generally sufficient [75]. However, a preliminary alum or PAC application optimization study will be ideal for each paper mill to determine the dosage, injection location, and the sizing efficiency. An excessive use of alum can lead to deposits on the paper machine, loss of sizing, and loss of sheet strength. Alum, PAC, or sodium aluminate deposits require downtime for cleanup; analysis has shown that these deposits are mainly aluminum hydroxide [Al(OH)_3_] and complex aluminum hydroxides [Al_x_(OH)_y_(SO_4_)nH_2_O, Al_x_(OH)_y_Cl_2_nH_2_O], which can be found on the primary screens, machine chest, headbox, blades, and vacuum box [73]. In addition, excessive use of alum has little effect on sizing at low concentrations but decreased sizing at higher concentrations. The overuse of alum has a degradative effect on paper as it becomes more acidic and undergoes acid hydrolysis that causes cellulose chain scission resulting in the loss of paper strength [8,52,68,70].

Finally, excessive use of defoamer and certain biocides has an adverse effect on sizing performance. It is known the excessive application of defoamer, especially at the wet end, destroys sizing [58,77,78], but it is not well understood if the defoamer or the biocide prevents ASA from anchoring to cellulose or if these chemicals just attack and ruin the sizing agent. Quaternary ammonium salt biocide is known to cause adverse effects on sizing while organosulfur and isothiazoline has almost no effect on sizing performance. More investigations are needed to determine the mechanism by which defoamers or biocides negatively affect ASA.

## 5. Conclusions

Several experiment-result papers and patents about the ASA application were reviewed from 1963 to 2020.

- It was believed that ASA formed covalent bonding with cellulose during the papermaking process. Nevertheless, most recent scientific evidence supports that the major sizing material in a sized paper is hydrolyzed ASA and its salts.
- The amount of covalent bonds between ASA and the fiber is very low and is not a requirement to sizing development.
- Hydrolyzed ASA contributes to the sizing performance; however, the direct application of hydrolyzed ASA to the pulp or the application of hydrolyzed ASA to the sheet leads to flocculation and does not achieve sizing.
- The pH, temperature, conductivity, and the ASA-emulsifier ratio are critical for ASA emulsion stability.
- Alum or PAC, when properly used improve sizing performance.
- The shelf life of the ASA emulsion is about 20 to 30 min. More work is needed to understand how the change in ASA concentration, ASA-starch ratio, temperature, and pH individually or collectively affects the ageing process.
- The use of novel chemistries to stabilize or extend ASA emulsion shelf life should be investigated for large scale availability, health and environmental impacts, and repercussions on the papermaking process.

## Data Availability

No new data were created or analyzed in this study. Data sharing is not applicable to this article.
